# A novel stemness classification in acute myeloid leukemia by the stemness index and the identification of cancer stem cell-related biomarkers

**DOI:** 10.3389/fimmu.2023.1202825

**Published:** 2023-06-19

**Authors:** Yue Huang, Zhuo Zhang, Meijuan Sui, Yang Li, Yi Hu, Haiyu Zhang, Fan Zhang

**Affiliations:** ^1^ Department of Biostatistics, School of Public Health, Harbin Medical University, Harbin, China; ^2^ National Health Commission (NHC) Key Laboratory of Cell Transplantation, The First Affiliated Hospital of Harbin Medical University, Harbin, China; ^3^ Department of Hematology, Southern University of Science and Technology Hospital, Shenzhen, China; ^4^ Key Laboratory of Hepatosplenic Surgery, Ministry of Education, The First Affiliated Hospital of Harbin Medical University, Harbin, China; ^5^ Medical Insurance Office, The First Affiliated Hospital of Harbin Medical University, Harbin, China; ^6^ Center for Bioinformatics, Faculty of Computing, Harbin Institute of Technology, Harbin, Heilongjiang, China; ^7^ Key Laboratory of Cardiovascular Disease Acousto-Optic Electromagnetic Diagnosis and Treatment in Heilongjiang Province, The First Affiliated Hospital of Harbin Medical University, Harbin, China

**Keywords:** AML, cancer stem cell, mRNAsi, biomarkers, SLC43A2

## Abstract

**Background:**

Stem cells play an important role in acute myeloid leukemia (AML). However, their precise effect on AML tumorigenesis and progression remains unclear.

**Methods:**

The present study aimed to characterize stem cell-related gene expression and identify stemness biomarker genes in AML. We calculated the stemness index (mRNAsi) based on transcription data using the one-class logistic regression (OCLR) algorithm for patients in the training set. According to the mRNAsi score, we performed consensus clustering and identified two stemness subgroups. Eight stemness-related genes were identified as stemness biomarkers through gene selection by three machine learning methods.

**Results:**

We found that patients in stemness subgroup I had a poor prognosis and benefited from nilotinib, MK-2206 and axitinib treatment. In addition, the mutation profiles of these two stemness subgroups were different, which suggested that patients in different subgroups had different biological processes. There was a strong significant negative correlation between mRNAsi and the immune score (r= -0.43, p<0.001). Furthermore, we identified eight stemness-related genes that have potential to be biomarkers, including SLC43A2, CYBB, CFP, GRN, CST3, TIMP1, CFD and IGLL1. These genes, except IGLL1, had a negative correlation with mRNAsi. SLC43A2 is expected to be a potential stemness-related biomarker in AML.

**Conclusion:**

Overall, we established a novel stemness classification using the mRNAsi score and eight stemness-related genes that may be biomarkers. Clinical decision-making should be guided by this new signature in prospective studies.

## 1 Introduction

Acute myeloid leukemia (AML) has an incidence of 4.3 per 100,000 adults aged over 15 per year and a 5-year survival rate of 24% (1). It is characterized by malignant clonal expansion of progenitor cells accompanied by differentiation arrest (2). There are two common causes of AML, including underlying hematological disorders and prior therapy (for example, exposure to topoisomerases II, alkylating agents, or radiation) (3). Based on their cytogenetic profile, AML patients can be classified into favorable, intermediate, and adverse risk groups, but the prognosis within these categories varies widely. In recent years, considerable progress has been made in developing treatments that are less burdensome for AML patients. Despite advances in supportive care, cytarabine- and anthracycline-based regimens and allogeneic stem cell transplantation remain the backbone of therapy. In addition, up to 50% of AML patients end up relapsing after receiving allo‐HSCT, while CAR‐T-cell treatment frequently fails due to lack of a specific AML antigen (4), antigen loss, or failure of CAR‐T-cell maintenance in AML patients. AML patients often experience resistance to chemotherapy due to the deregulation of apoptosis, especially in stem cells (5). There are increasing numbers of studies demonstrating that AML stem cells are at the root of relapses and multidrug resistance in the disease diagnosis and classification (6–8). By arresting in the G0 phase, cancer stem cells can cause cancer relapse, metastasis, multidrug resistance (9), and radiation resistance. Cancer stem cells are believed to play a crucial role in cancer relapse, and eliminating them is crucial to cancer treatment.

In recent years, cancer stem cells have been isolated from a variety of solid tumors, including breast carcinoma (10, 11), brain (12, 13), pancreatic (14, 15), colon (16, 17), head and neck (18), hepatic (19), lung (20), prostate (21), bladder (22), ovarian malignancies (23), melanoma (24) and musculoskeletal sarcomas (25). There is no doubt that cancer stem cells are different from tumor bulk cells in terms of their properties, and researchers agree that these cells represent a population of cancer cells with specific properties. The biological activities of cancer stem cells are regulated by several pluripotent transcription factors, such as OCT4, Sox2, Nanog, KLF4, and MYC (26). A one-class logistic regression (OCLR) machine learning algorithm was used to calculate stemness indices to evaluate the degree of oncogenic dedifferentiation (27). Tian et al. found that mRNAsi value was higher in head and neck squamous cell carcinoma tissues than in normal tissues (28). Zhang et al. found that the lower mRNAsi group had a better 5-year overall survival in major lung adenocarcinomas (LUADs), and they screened several stem cell biomarkers (29). Wang et al. classified hepatocellular carcinoma patients into three different molecular subtypes based on 212 mRNAsi-related genes (30). Qin et al. identified 5 mRNAsi-related genes that were highly expressed in tumor samples compared to normal samples (31). Stem cell therapy, which involves all procedures using stem cells, has emerged as a promising option for cancer treatment.

Stem cells in AML have been shown to be the basis for self-renewal, quiescence in the cell cycle, and resistance to chemotherapy (32). A clinical perspective indicates that treatment courses must eliminate stem cells for the disease to be eradicated and long-term remission to be achieved (33). Patients with a higher frequency of stem cells had a poorer prognosis with a shorter relapse-free survival (34). From diagnosis to relapse, the leukemic stem cell frequency increased 9- to 90-fold. This further suggests that AML stem cells may play an important role in the treatment strategy. Carolina reported that the IL8-CXCR2 pathway is frequently dysregulated in AML and MDS stem cells and can serve as a novel therapeutic target in these diseases (35). Carsten reported that CD70/CD27 signaling promotes blast stemness and is a viable therapeutic target in AML (36). In addition, stem cells have been shown to display high plasticity, which changes their phenotypic appearance and functions. The tumor microenvironment can be altered by chemo- and radiotherapeutics, as well as senescent tumor cells. However, limited studies have been carried out to systematically analyze AML stem cells based on high-dimensional omics data. In order to guide clinical decision-making, precision biomarkers are a basic scientific problem that needs to be solved urgently.

In this research, the stemness index of AML patients was evaluated by transcriptome analysis. All AML patients were divided into two subgroups, stemness subgroups I and II, based on 143 mRNAsi-related genes by consensus clustering. Different mutations, clinical features and survival outcomes have been reported in different stemness subgroups. Eight mRNAsi-related genes were identified, and most genes were negatively related to the mRNAsi score. The key genes were validated in the test set and demonstrated stemness biological functions. Furthermore, we recommend nilotinib for patients in stemness subgroup I based on the half-maximal inhibitory concentration (IC50) value calculation. In conclusion, our research provides a novel direction of stemness therapy for AML and guides clinical decisions for AML patients.

## 2 Method

### 2.1 Multiomic data acquisition and patient population

We downloaded the AML gene expression data and corresponding clinical information and follow-up data from The Cancer Genome Atlas (TCGA). We defined the TCGA-AML dataset as the training set. In addition, we obtained other AML gene expression and clinical data from the Beat program (37), and we defined the Beat-AML dataset as the test set. Finally, we enrolled 151 TCGA-AML and 451 Beat-AML samples in our study. All expression data were converted to transcripts per million (TPM) for downstream analyses. In **Table 1**, we present the demographics and follow-up data for the two cohorts with AML. Additionally, the whole-exome sequencing data (MAF format) of 151 AML patients were downloaded from the TCGA database.

**Table 1:** Demographics and clinicopathological features of AML patients.

	TCGA-AML (N=151)	Beat-AML (N =451)
Age (mean (SD))	54.17 (16.07)	56.96 (17.97)
Sex (male (%))	83 (55%)	258 (57.2%)
Dead (%)	97 (64.2%)	238 (52.8%)
Survival Time(mean (SD))	375.6 (388.91)	419.56 (466.75)

### 2.2 Differential analysis of mRNAsi levels in the high versus low stemness groups

The mRNAsi was calculated by the gene expression profiles of normal pluripotent stem cells (PSCs), including PSCs and embryonic stem cells (ESCs), which were collected by the Progenitor Cell Biology Consortium (PCMC, https://progenitorcells.org/). First, the 78×8087 stemness-related matrix for 78 stem cell samples and 8087 protein-coding genes was obtained, and the expression data were centered by the mean. Second, we acquired the stemness signature by the OCLR algorithm using the glmnet R package, which is a machine learning algorithm used to extract transcriptomic and epigenetic feature sets derived from nontransformed pluripotent stem cells and their differentiated progeny. Third, the Spearman correlations between the weight vectors of the stemness signature and mRNA expression were determined. Finally, we transformed the stemness index to the range (0–1) through the following equation:


(1) $$\begin{array}{c} x=\frac{x-x_{min}}{x_{max}-x_{min}} \end{array}$$

Then, we obtained the mRNAsi for each AML sample, including the TCGA-AML and Beat-AML cohorts.

AML patients in the TCGA were classified into high stemness and low mRNAsi subgroups based on their median mRNAsi scores. We obtained the differentially expressed genes (DEGs) between the high and low mRNAsi subgroups by the limma R package. False positives were corrected using Benjamini−Hochberg false discovery rate (FDR)-adjusted P values. The DEGs were determined using an FDR of 0.01 and a fold change (FC) > 2. Then, we completed Gene Ontology (GO) pathway analysis using the DEGs.

### 2.3 Stemness-based molecular classification

We identified the molecular classification of AML patients according to the expression of the DEGs, which was carried out using unsupervised consensus clustering by the ConsensusClusterPlus R package (38). Clustering was performed using 1000 iterations, and 80% of the data were sampled for each iteration. Two stemness subgroups were identified based on the cumulative distribution function (CDF) curves. We named these two subgroups stemness I and stemness II.

### 2.4 Identifying the immune-based molecular classification

The enrichment analysis was quantified using single-sample gene set enrichment analysis (ssGSEA) (39) based on 29 immune signatures (40). Similarly, we identified three immune-related subgroups based on the ssGSEA score using unsupervised hierarchical clustering. Then, we calculated the stromal score, immune score and ESTIMATE score using the Estimation of Stromal and Immune cells in Malignant Tumor tissues using Expression data (ESTIMATE) (41), which is a method that uses gene expression signatures to infer the fraction of stromal and immune cells in tumor samples. We also compared the tumor-infiltrating immune cells (TIICs) among the three immune-related subgroups, and CIBERSORT was used to quantify the compositions of 22 types of TIICs Robust enumeration of cell subsets from tissue expression profile (42).

### 2.5 Construction and validation of the stemness subtype predictor using multiple machine learning algorithms

As mentioned before, we used the TCGA-AML dataset as the training set and the Beat-AML dataset as the test set. First, we selected the most important genes using least absolute shrinkage and selection operator (LASSO) regression, random forest and Boruta (RF), and extreme gradient boosting (XGBoost) analyses based on the training set. In this training process, the expression of the DEGs was used as the input data, and the outcome was the binary variable that represented the two stemness groups separately. Then, we took the intersection of these three important gene sets to obtain the final list, which was the most important stemness-related gene set and was visualized by a Venn plot. For these genes, we calculated the Pearson correlation coefficients with mRNAsi, IC50, MMR genes and RNA modulations. Finally, multivariable logistic regression analysis was performed on the key genes to construct and test the predictive model, and the areas under the receiver operating characteristic (ROC) curve (AUC) were used to evaluate the performance of the three machine learning methods.

### 2.6 The prediction of drug response and survival analysis

The pRRophetic R package was used to predict the chemotherapeutic response, and the IC50 was used as a measure based on the Genomics of Drug Sensitivity in Cancer (GDSC) database (43, 44). In addition, multivariable survival analysis was performed to evaluate the prognosis of patients in different stemness subgroups, and we adjusted for covariates including age, morphology and sex.

## 3 Results

### 3.1 Identification of two stemness subgroups with DEGs

**Figure 1:**
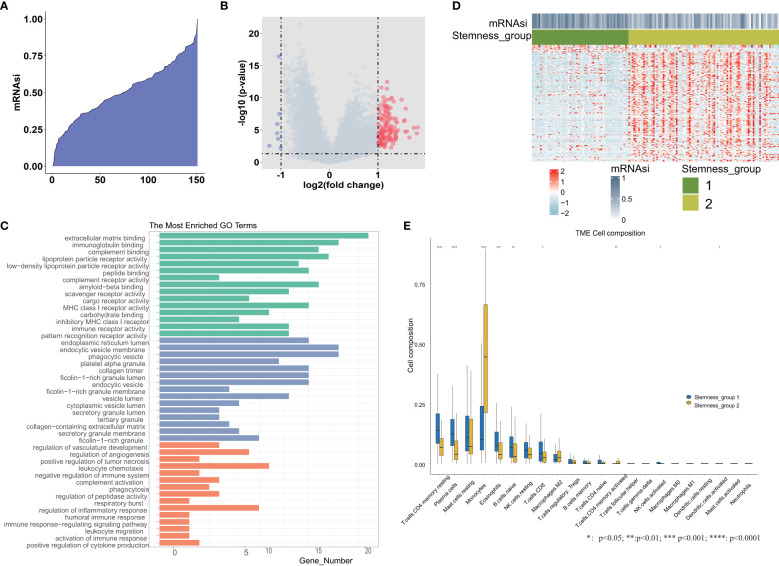
Differential expression analysis between the high and low mRNAsi groups. **(A)** An overview of mRNAsi in TCGA-AML. Columns represent samples ranked by mRNAsi from low to high (top row). **(B)** Identification of DEGs between the mRNAsi high and low groups according to the median value. **(C)** Functional enrichment analyses of DEGs, including significantly enriched biological processes, cellular components, and molecular functions. **(D)** Heatmap of the expression patterns of 143 DEGs, with red indicating high expression and blue indicating low expression. **(E)** The tumor immune microenvironment cell composition difference between stemness group I and stemness group II. *, **, *** and **** represent the P value <0.05, 0.01, 0.001, 0.0001 respectively.

We calculated the mRNAsi value by the OCLR algorithm, which was based on the gene expression and PSCs in the training set. These 151 mRNAsi values were ranked in ascending order, and the median value of mRNAsi was used as the threshold (**Figure 1A**). According to the threshold, all TCGA-AML patients were divided into two groups: high and low mRNAsi groups. Then, differential expression analysis between these two groups was performed. A total of 143 DEGs were identified, including 7 downregulated and 136 upregulated genes. We plotted the volcano map for these DEGs (**Figure 1B**). The GO functional enrichment analysis of these DEGs was performed by the enrichGO function (**Figure 1C**), including extracellular matrix binding, immunoglobulin binding, phagocytic vesicle, collagen trimer, regulation of angiogenesis and respiratory burst. Based on 143 DEGs, we identified stemness subgroups I and II by unsupervised consensus clustering (**Figure 1D**). There were 59 patients in stemness subgroup I, with the remaining 92 patients in stemness group II. The heatmap showed that the different stemness subgroups have their own unique patterns of expression. We also found immune cell composition differences between stemness group I and stemness group II (**Figure 1E**).

### 3.2 Different stemness subgroups showed distinct mutation and survival patterns

**Figure 2:**
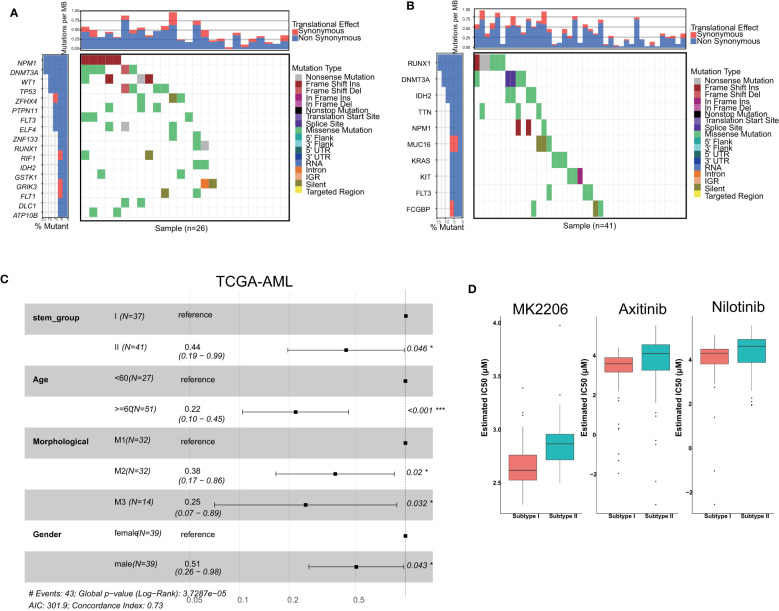
Comparisons of somatic variations and survival between stemness subtypes I and II. Waterfall plots showing the top mutations in stemness subtypes I **(A)** and II **(B)**. **(C)** Forest plot of survival analysis. **(D)** Boxplots indicated that patients with stemness subtype I were more sensitive to three drugs (nilotinib, MK2206 and axitinib) than those with stemness subtype II.

As previously mentioned, all TCGA-AML samples were categorized into two groups, which were named stemness subgroup I and stemness subgroup II. As shown in **Figures 2A, B**, the different stemness subgroups showed distinct mutation patterns. For the patients in stemness subgroup I, NPM1, which is the gene for nucleophosmin and belongs to the nucleophosmin/nucleoplasmin family of proteins (45), was the most frequently mutated gene. RUNX1, which is a transcription factor that is widely expressed in hematopoietic cells and indispensable for the establishment of definitive hematopoiesis (46), was the most frequently mutated gene in stemness subgroup II. DNMT3A was the second most frequently mutated gene in stemness subgroups I and II; DNMT3A encodes a DNA methyltransferase and was independently associated with a poor outcome (47). In addition, these two stemness subgroups showed their own specific mutation patterns, which consisted of different mutated genes. Considering the effect of age, morphology and sex on survival, we performed survival analysis after adjusting for these covariances. There was a significant survival difference between these two stemness subgroups after correcting for other confounding factors (**Figure 2C**). However, when we performed survival analysis based only on patients in the M1, M2 and M3 groups, the results indicated that the TCGA-AML patients in stemness subgroup II presented significantly better overall survival (p = 0.046) than those in stemness subgroup I. The median overall survival time in stemness subgroup II patients was 396 days, which was longer than that of stemness subgroup I patients (365 days). This result suggested that mutations may rewire tumor development, which in turn changes their prognosis.

### 3.3 The patients in stemness subgroup I were sensitive to nilotinib

We screened candidate chemotherapy drugs for AML patients using the pRRophetic R package in the training set. The IC50 value was estimated for each drug for individual patients. The IC50 value was inversely related to drug sensitivity. Finally, we found that the IC50 values of nilotinib were significantly lower in patients in stemness subgroup I (**Figure 2D**), which indicated that patients in subgroup I were more sensitive to nilotinib than patients in stemness subgroup II. The Beat-AML study provided drug response information, so we analyzed the IC50 value directly. We found that patients in subgroup I were more sensitive to nilotinib, which was consistent with the results in the training set. Therefore, nilotinib is expected to become the specific drug for patients in stemness subgroup I. Similarly, MK-2206 and axitinib were more suitable for patients in stemness subgroup I.

### 3.4 Association between the stemness subgroups and tumor immune microenvironment

**Figure 3:**
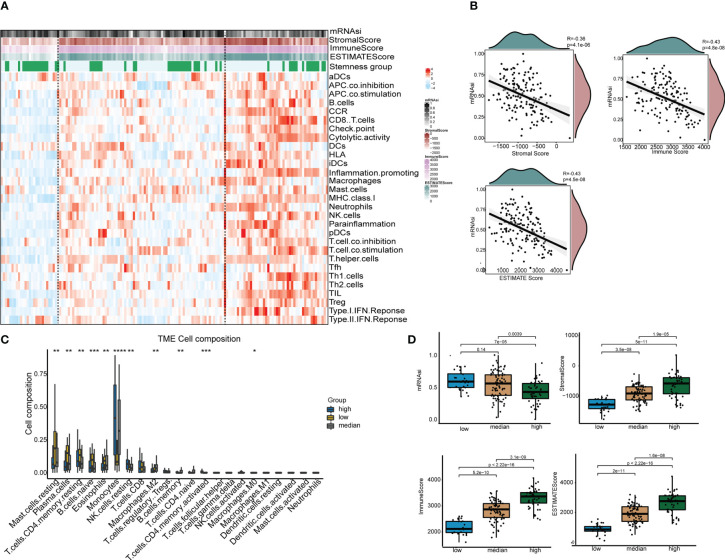
The tumor immune microenvironment patterns and immunogenomic features of AML associated with mRNAsi. **(A)** The immune subtypes of patients were categorized on the basis of the overall immune activity of AML. **(B)** Correlation analyses between mRNAsi and the stromal score, immune score, ESTIMATE score and tumor purity evaluated by the ESTIMATE algorithm. **(C)** Comparisons of the abundances of 22 immune cells in three immune subtypes. ∗ means P<0.05, ∗∗ means P<0.01, ∗∗∗ means P<0.001, and ∗∗∗∗ means P<0.0001. **(D)** Comparisons of mRNAsi, the infiltration level of stromal and immune cells and the ESTIMATE score in different immune subtypes by boxplots.

To identify the immune-related subgroups, we obtained the enrichment score of 29 immune signatures representing the overall immune activity using ssGSEA. All TCGA-AML patients were divided into three immune-related subgroups: high immunity group (48 patients), median immunity group (77 patients) and low immunity group (26 patients). As **Figure 3A** shows, the patterns of these three immune subgroups were distinct. To understand the full picture of the immune system, we evaluated the microenvironmental components using the ESTIMATE and CIBERSORT methods. The Pearson correlation between immune and stemness is shown in **Figure 3B**. The correlation value between mRNAsi and the stromal score was -0.36 (p<0.001), and a similar trend was found in the correlation between mRNAsi and the immune score (R = -0.43, p<0.001). Thus, these results suggest a negative relationship between stemness and immunity. Interestingly, the relationship between mRNAsi and the ESTIMATE score was in agreement with previous findings. In addition, we compared the abundances of 22 types of immune cells among the three immune subgroups (**Figure 3C**). We found that there were several immune cell type differences in these immune subgroups, including mast cells, plasma cells, CD4 T cells, native B cells, eosinophils, monocytes, NK cells, macrophages and memory B cells. Once more, the results demonstrated that the immune subgroups represented distinct immune activities and cells. As shown in **Figure 3D**, the distributions of the mRNAsi score, stromal score, immune score and ESTIMATE score were significantly different among the different immune-related subgroups. The association between immunity and stemness may provide a combination treatment strategy for AML patients.

### 3.5 Construction and validation of the stemness subgroup classification

**Figure 4:**
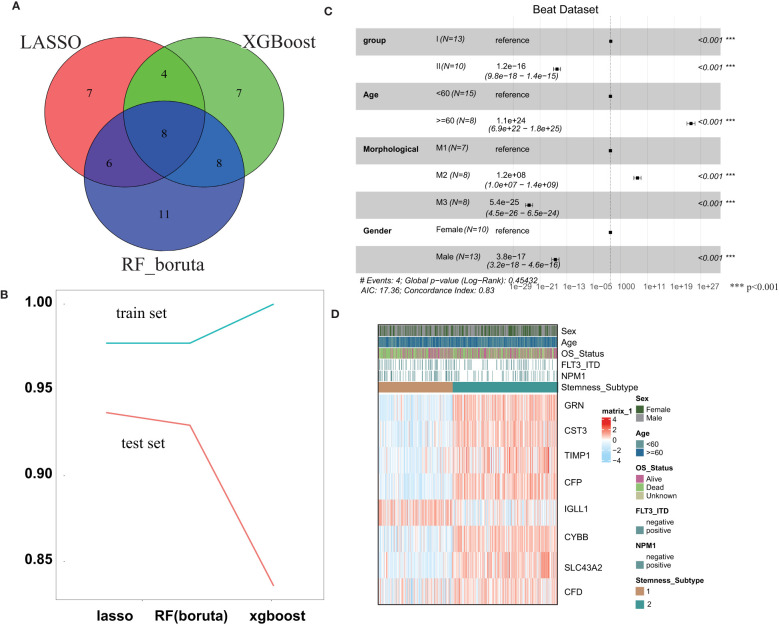
Construction and validation of the Stemness Subtype Predictor. **(A)** Venn diagram identified the seven most critical stemness subtype-specific genes that were shared by three feature selection algorithms. **(B)** The performances of three machine learning algorithms (LASSO, RF, and XGBoost) for feature selection were evaluated in the training set and test set. AUCs were generated by ROC analysis. **(C)** Forest plot of survival analysis. **(D)** Beat RNA sequencing data were used to further validate the clinical application value of the stemness-based classification, which was visualized by heatmaps. *** represent the P value < 0.001.

In the training set, three machine learning methods, including LASSO, RF and XGBoost, were employed to identify the key genes that play important roles in stemness based on the 143 screened DEGs. These three machine learning methods identified 25, 33 and 27 genes separately (**Figure 4A**), and the final 8 key genes were the intersection of these three gene lists. To explore the performance of the three machine learning methods, the AUC was used to measure the feature selection capability (**Figure 4B**). The AUC was greater than 0.975 regardless of which methods were employed in the training set, and this value dropped in the test set but was still approximately 0.9. The results of multivariable survival analysis in the test set demonstrated that patients in stemness subgroup II had better overall survival than those in stemness subgroup I (p<0.001) (**Figure 4C**). This result was consistent with the survival analysis result in the training set. Therefore, our stemness classification could bring clinical benefit for AML patients. By plotting the key gene expression profile in the test set, the stemness subgroups were clearly present. These results confirmed that the 8 key genes indeed play a large role in the stemness process (**Figure 4D**).

### 3.6 Analysis of the most important genes associated with stemness

**Figure 5:**
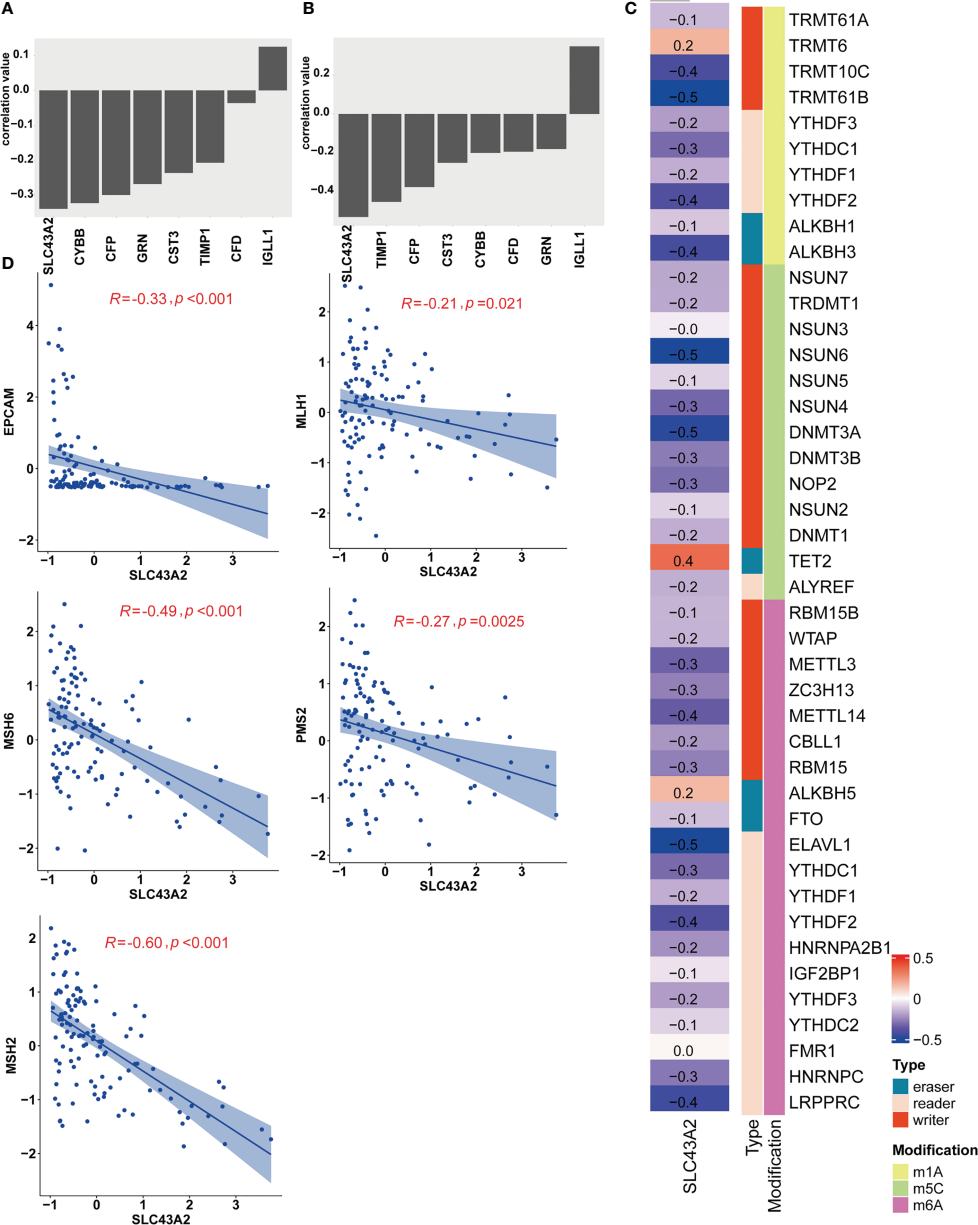
SLC43A2 plays an important role in tumorigenesis. The correlation values between SLC43A2 and nilotinib IC50 **(A)** and mRNAsi **(B)**. **(C)** The heatmap shows the correlation between SLC43A2 expression and RNA modulations in AML. **(D)** The scatter plot displays the associations between SLC43A2 and 5 MMR genes in AML.

For the 8 key genes that play an important role in the stemness process, we calculated the Pearson correlation value between these genes and the IC50 value of nilotinib. We aimed to investigate the effect of these 8 stemness-related genes on the response to nilotinib treatment and to identify their potential biological functions. As shown in **Figure 5A**, most genes were inversely related to the IC50 value of nilotinib, including SLC43A2, CYBB, CFP, GRN, CST3, TIMP1 and CFD. The correlation value between SLC43A2 and the IC50 value of nilotinib was -0.34, which demonstrated that SLC43A2 is associated with nilotinib sensitivity. Interestingly, only IGLL1 showed a positive correlation value (0.12) with the IC50 value of nilotinib. Then, we also plotted the Pearson value between these genes and mRNAsi, and we found a similar trend (**Figure 5B**). Notably, SLC43A2 also exhibited the strongest relationship with mRNAsi, which further demonstrated the important function of SLC43A2. Next, we investigated the correlation between SLC43A2 and RNA modulator gene expression. Surprisingly, we discovered that high SLC43A2 expression was associated with a majority of RNA modulator genes in the TCGA-AML dataset, including m1A, m5C and m6A (**Figure 5C**). Interestingly, most of the relationships were negative except for TRMT6, TET2 and ALKBH5. Next, we discovered that SLC43A2 was negatively correlated with multiple mismatch repair (MMR) genes in AML (**Figure 5D**), which indicates that SLC43A2 was affected by MMR. Thus, SLC43A2 was expected to be a potential stemness-related biomarker in AML.

## 4 Discussion

Previous studies have investigated the risk stratification of the stemness index in clinical cohorts with multiple cancers. However, the stemness index has not been explored in terms its comprehensive prognostic value in AML. Furthermore, no studies have investigated the functional annotation of stemness index-associated genes. In our study, we identified two stemness subgroups based on gene expression data by consensus clustering. The survival analysis demonstrated that patients in stemness subgroup I had a poor prognosis. In addition, we identified eight mRNAsi-related genes (SLC43A2, CYBB, CFP, GRN, CST3, TIMP1, CFD and IGLL1) using three machine learning methods, including LASSO, XGBoost and RF. Most of the key genes were negatively related to the mRNAsi score, especially SLC43A2, for which the correlation coefficient with mRNAsi was -0.39. Nilotinib was suggested to be the most appropriate candidate for the treatment of patients in stemness subgroup I. These results suggested that the stemness profile could benefit AML patients.

SLC43A2 is a methionine transporter that tumor cells express at high levels, allowing them to monopolize methionine consumption. By inhibiting tumor SLC43A2 genetically and biochemically, T cells were able to restore H3K79me2, thereby boosting spontaneous and checkpoint-induced immunity (48). That is, SLC43A2 should be negatively correlated with immunotherapy response. In our study, we found that SLC43A2 was negatively correlated with the mRNAsi score. SLC43A2 has dual roles in the combination treatment of immunotherapy and stemness therapy. Researchers have found that cancer stem cells accelerate tumor recurrence and radiotherapy and chemotherapy resistance. AML patients can benefit greatly from immunotherapy, which is a powerful anticancer treatment. One important concern when considering combination therapy is the prediction of benefit, especially when potentially combining immunosuppressive therapies and stemness therapy. Shi et al. constructed a five-gene signature based on tumor stemness and immune-related specific genes to predict the response to radiotherapy or immunotherapy and relapse in LUAD (49). Furthermore, for the feasibility of the synthesis of such drugs, the dual identity of the drug target should be considered. In addition, we found a significant difference in the two classification types of immune and stemness, which further suggested that combination therapy can cure AML patients.

Axitinib, MK-2206 and nilotinib were the most appropriate drugs for patients in stemness subgroup I. Axitinib is being tested in phase III trials for solid carcinomas and inhibits VEGFR-1, -2, and -3 (50). Saha reported that axitinib exerts direct cytotoxic activity against a number of patient-derived glioblastoma stem cells (51). The AKT inhibitor MK-2206 decreased cell proliferation in CRC cells, resulting in a significant reduction in stemness (52). David reported that the combination of the Hedgehog pathway inhibitor LDE225 and nilotinib eliminates stem and progenitor cells (53). Hence, these three drugs could act in stem cells, albeit not necessarily acting on the same drug targets. The patients in stemness subgroup I had higher mRNAsi values, which means that they had more cancer stemness capability. All three drugs were recommended for stemness subgroup I, and the results of the drug analysis were logically reasonable.

Nevertheless, there are still several limitations in our study. First, to validate this study, only one cohort with AML patients was included, and the representativeness of the data was not strong. In the future, we need to expand the sample size from our own center. Second, due to the limitation of the available data, we aim to establish an AML cohort to test the combination response of immunotherapy and stemness therapy. In conclusion, we developed a novel stemness classification for AML patients using consensus clustering to guide clinical treatment. Eight biomarker genes were found to be closely related to AML stem cell characteristics, and seven genes were negatively related to the mRNAsi score. SLC43A2 is expected to be a potential stemness-related biomarker in AML.

## Data availability statement

The original contributions presented in the study are included in the article/supplementary materials, further inquiries can be directed to the corresponding author/s.

## Author contributions

YuH and ZZ analyzed the data and drafted the manuscript. MS, YiH and YL downloaded the data and prepared the figures. FZ and HZ designed the research and edited the manuscript. All authors reviewed, revised, commented on and approved the final manuscript. All authors contributed to the article and approved the submitted version.
